# Contact Profiles in Eight European Countries and Implications for Modelling the Spread of Airborne Infectious Diseases

**DOI:** 10.1371/journal.pone.0005931

**Published:** 2009-06-17

**Authors:** Mirjam Kretzschmar, Rafael T. Mikolajczyk

**Affiliations:** 1 Centre for Infectious Disease Control, RIVM, Bilthoven, The Netherlands; 2 Julius Centre for Health Sciences and Primary Care, University Medical Centre Utrecht, Utrecht, The Netherlands; 3 School of Public Health, University of Bielefeld, Bielefeld, Germany; Columbia University, United States of America

## Abstract

**Background:**

For understanding the spread of infectious diseases it is crucial to have knowledge of the patterns of contacts in a population during which the infection can be transmitted. Besides contact rates and mixing between age groups, the way individuals distribute their contacts across different locations may play an important role in determining how infections spread through a population.

**Methods and Findings:**

Representative surveys were performed in eight countries to assess the number of social contacts (talking to another person at close distance either with or without physical contact), using a diary approach in which participants recorded individual contacts. The overall sample size was 7290 respondents. We analyzed the reported numbers of contacts per respondent in six different settings (household, work, school, leisure, transportation and others) to define different contact profiles. The identification of the profiles and classification of respondents according to these profiles was conducted using a two-step cluster analysis algorithm as implemented in SPSS.

We identified seven distinct contact profiles: respondents having (1) mixed: contacts predominantly at school, during transportation and leisure time, (2) contacts during leisure time, (3) contacts mainly in the household (large family), (4) contacts at work, (5) contacts solely at school, (6) contacts in other places and finally (7) respondents having a low number of contacts in any setting. Similar contact profiles can be found in all eight European countries which participated in the study. The distributions of respondents across the profiles were similar in all countries. The profiles are dominated by work, school and household contacts. But also contacts during leisure activities play an important role in the daily lives of a large fraction of individuals. A surprisingly large number of individuals has only few contacts in all locations. There was a distinct age-dependence in the distribution of the population across contact profiles.

**Conclusions:**

In contrast with earlier studies that focussed on the contribution of different age groups to the spread of an infectious disease, our results open up the opportunity to analyze how an infection spreads between locations and how locations as work or school are interconnected via household contacts. Mathematical models that take these local contact patterns into account can be used to assess the effect of intervention measures like school closure and cancelling of leisure activities on the spread of influenza.

## Introduction

Contacts between individuals are instrumental for the direct transmission of many infectious diseases. Recently, increased effort has been put into measuring the numbers and characteristics of contacts that lead to the transmission of airborne infections like influenza [1]–[6]. Although it is not known with certainty what type of contact between two individuals is sufficient for transmission of a pathogen, it has been shown that conversational contacts or social contacts in close proximity are a good proxy for contacts leading to transmission [5]. Quantitative information about these contacts is therefore needed to inform mathematical modelling that is used to analyse and evaluate intervention strategies and contingency planning [7]–[11]. Up to now the main focus of these measurements was on the numbers of contacts per day between different age groups. However, characteristics of contacts may also influence the way an infection spreads through a population, for example the place where contact occurs, or the proximity of contact. Additionally, it can be of interest, how individuals distribute their contacts across different locations, as opposed to overall distributions of contacts for the entire population across locations. A large study to collect this type of information in representative samples of the populations of eight European countries was conducted recently (POLYMOD project) [4]. Average numbers and duration of contacts and age mixing matrices for these countries have been reported elsewhere.

Traditionally, mathematical modelling of the spread of airborne infectious diseases used age-mixing matrices that were chosen for mathematical convenience, such as proportionate mixing and so-called “who acquires infection from whom” (WAIFW) matrices [12]. However, age - albeit important – is most certainly not the only variable that has a major impact on the mixing patterns in a population. Other variables, such as location and setting (home, school, work etc), in which a contact occurs, are influential in determining who has contact with whom. Also, contacts taking place in different settings might be of different intensity and or proximity as was shown earlier in [2], [13]. More importantly, the typical distribution of the contacts of an individual across locations might be influential and cannot be accounted for by average contact rates among populations groups. Information about mixing in different settings is important for the analysis of vaccination strategies for young children and adults, respectively, who distribute their contacts in different ways across settings and therefore might be exposed to infection risks from different sources. Ideally, one would like to know how many contacts children and adults have within their households and outside of households in other locations. In other words, we are interested in how individuals distribute their contacts across various locations/settings and in how those contacts differ in duration and proximity. In the following we will use the term “contact profile” to refer to a distribution of contacts of a single person across a number of different locations/settings.

We report on an analysis of the contact data collected in the POLYMOD study using cluster analysis techniques. Our aim is to identify typical contact profiles which were displayed by respondents in the samples from the eight countries, characterize those profiles and compare the countries with respect to the distribution of different contact profiles. We then discuss how these results might be used to further develop mathematical modelling of the spread of airborne infectious diseases.

## Methods

### Survey sample

The dataset used here has been extensively described and analysed in Mossong et al [4]. In brief, cross-sectional surveys of contact patterns were conducted in Belgium (BE), Germany (DE), Finland (FI), Great Britain excluding Northern Ireland (GB), Italy (IT), Luxembourg (LU), the Netherlands (NL) and Poland (PL). In all countries samples representative for the general population were aimed for with an oversampling of children of 0–5 years of age. The data collection was organized for each country separately and slightly different methodologies were employed: in 6 countries random digit dialling was used for the recruitment of participants, in two countries recruitment was performed face-to-face (random walking route) either as a separate study (PL) or as a part of a larger multi-theme survey (DE). Participants in NL and FI were recruited via population registers. The surveys were performed between May 2005 and September 2006 with oral informed consent of the participants. In NL the survey is part of a larger ongoing serological study and the partial sample provided for this analysis was smaller than for other countries. Participants received a self-administered questionnaire in form of a diary and were asked to fill in each contact made during the course of one day. Some information about the contact person (age or age range, gender) or contact (where the contact occurred, how often contacts with this person occur in general, whether the contact involved physical contact, and the duration of contact) was also collected. Additionally, basic socio-demographic information about the participant was obtained. Since the study covered all age groups three or two different versions of the questionnaire were used: in all countries but Germany a questionnaire for children (0–14 years), adolescents (15–18) and adults (19+); in Germany only two versions of the questionnaire were used: for children or young adolescents (0–14) and for older adolescents or adults (15+). Questionnaires for young children were filled in by parents or guardians. Informed consent was obtained prior to the distribution of the questionnaire.

### Variables used in this analysis

A contact was defined either as a two way conversation in close proximity or a physical contact like shaking hands or kissing. The place of contact was recorded in six different categories or settings: school, work, home, in transportation, during leisure activities and in other places. The last category was not further specified and no information was obtained as to what “other places” might refer to. In some cases contacts with one and the same person were recorded in different settings. For the purposes of this analysis we counted that contact person only once. To attribute the contacts with that person to a specific setting, we used the following hierarchy: contacts at home > contacts at work > contacts at school > contacts in leisure time > contacts in other place > contacts in transportation. The hierarchy was based on the putative duration of contacts in the given settings. In Germany, Netherlands and Belgium if participants had many contacts at work or at school (>10 in NL and DE and >20 in BE) they were asked not to record them separately in the diary but to only provide a general estimate of the number of those contacts. These numbers were then added to either the numbers of work or school contacts for those participants who had filled in a diary on a working day. Since some respondents reported excessively large numbers in this aggregate category of work/school contacts, we limited the numbers to a maximum of 50 for this analysis.

### Statistical analysis

We performed a cluster analysis on the data set defined by the numbers of contacts in different settings for every survey respondent. More formally, the numbers of contacts in each of the 6 settings for a respondent defines a vector (n_1_,…, n_6_) in a 6-dimensional non-negative cone. A cluster analysis was performed to find clusters in that space. We used the two-step cluster algorithm that is implemented in SPSS with all variables treated as continuous, a distance measure based on likelihood ratio, and auto-clustering used to identify the optimal number of clusters in the analysis. The first step of the two-step algorithm is a BIRCH algorithm to define pre-clusters [14], in the second step these preclusters are joined to clusters using an agglomerative hierarchical algorithm [15], [16]. The number of clusters can be fixed or can be determined by the clustering procedure. In the latter case, the number of clusters is determined on the basis of the Bayesian Information Criterion (BIC) or the Akaike Information Criterion (AIC). The algorithm based on continuous variables and likelihood ratio as distance measure assumes a multivariate normal distribution of the data, but there is some evidence that the procedure remains valid under violation of the distribution assumption [16]. While in our sample the distribution of some of the clusters might have a large mass at zero for some of the 6 variables, this distribution can be viewed as a degenerate normal distribution with mean and standard deviation of zero in these variables.

Given the differences across countries in relation to mean contact numbers [4] we performed the cluster analysis for each country separately. During the first step of pre-clustering of cases into small sub-clusters the initial ordering of records influences the resulting cluster distribution, because the sub-clusters are not resolved during the further analysis. We therefore performed the cluster analysis 100 times for each country with varying initial ordering of records and allowed the number of clusters to vary in each run and each country. In some countries this resulted in up to 10 separate clusters, but 7 clusters or less were more common (Table 1). Given that all the results were based on the same data, this variation shows that statistical criteria regarding the number of clusters can provide only a limited guidance in this case. Based on the fact that for some countries a seven clusters solution was favoured by the algorithm, we fixed the number of clusters to seven in all countries. These seven clusters also provided reasonable interpretation (see below). The purpose of the further analysis was to demonstrate that partitioning into 7 clusters results in similar contact profiles (defined as a typical distribution of contacts across different settings) across countries and thus country invariant information can be derived.

**Table 1:** Distribution of the optimal number of clusters in 100 runs of cluster analysis with changing ordering of cases based on statistical criteria.

Number of cluster	BE	DE	FL	GB	IT	LU	NL	PL
2	60	30	9	57	3	51		47
3	12	1	12		6	16		5
4	1	39	4	1	4	8	100	16
5	19	21	2	4	6	3		11
6		3	6	4	14	13		
7	8	1	49	34	65	6		4
8		4	17		2	2		7
9		1	1					2
10						1		

Taking into account the ordering effect, after setting the number of clusters in each country to 7 we performed another 100 runs of the cluster analysis for each country. For all countries, the resulting clusters displayed similar properties. Based on these properties we defined 7 distinct contact profiles (Table 1). A contact profile P_j_ is defined as a vector M_j_ = (m_1j_,…, m_6j_) where m_ij_ is the median of {n_ij_^k^, k belongs to cluster P_j_}. We distinguish the contact profiles P_1_,…, P_7_, which are characterized by their vector M_j_. For some P_j_ there is a dominant m_ij_, for others two or more m_ij_ characterize the distribution (Table 2). These contact profiles could then be interpreted in terms of their overall numbers of contacts, their main contact location, and the proportion of contacts in other locations.

**Table 2:** Definition of the contact profiles: For every cluster the distribution of (n_1_,…, n_6_) was determined based on all respondents in that cluster.

Profiles	Characterization of the cluster defining the profile
*Professional*	contacts at work have highest median as compared to other locations: m_11_ = max {m_1i_∶i = 1,…,6}
*School*	contacts at school have highest median m_22_ = max {m_2i_∶i = 1,…,6}
*Leisure*	contacts during leisure activities have highest median: m_33_ = max {m_3i_∶i = 1,…,6}
*Big home*	contacts at home have highest median: m_44_ = max {m_4i_∶i = 1,…,6}
*Other place*	contacts in “other place” have highest median: m_55_ = max {m_5i_∶i = 1,…,6}
*Mixed*	contacts in more than one location have a median higher than the remaining cluster: m_6i_>m_7i_ for at least two i = 1,…,6.
*Low contacts*	The remaining cluster

Every cluster j was then characterized by a vector of medians of these distributions M_j_ = (m_j1_,…,m_j6_), j = 1,…7. Based on these medians the clusters were assigned as described in the table starting at the top and going down.

When performing the cluster analysis with a fixed number of clusters some of the profiles could not be identified in each run – this occurred when two or more of the clusters were classified as the same profile based on definitions given in Table 1. While refining the definitions in Table 1 could help in these cases, we decided to maintain the simple definitions and accept the reduced number of profiles. Additionally, some respondents were attributed to different profiles in different runs. To test the stability of the profile attribution of individual respondents we evaluated how often respondents were attributed to the same profile in the 100 simulation runs of the cluster analysis. While less than 10% of respondents were attributed to the same profile in only 50% of runs, around 70% of respondents were classified in the same profile in more than 80% of runs and around 50% in more than 90% of runs. The most frequently occurring profile for each respondent was then defined as the contact profile for that respondent.

## Results

### The sample

A total of 7290 participants recorded 97904 contacts. The characteristics of the sample were published in Table 2 of [4]. In brief, there was a slightly higher proportion of female participants, especially in Germany. Single person households were most common in Germany and least common in Italy and Poland. Italy and Poland also had the highest proportion of households with 4 or more persons. In most countries less than the expected 28.5% diaries were filled in for weekend days, and that percentage was especially low in Germany and Luxemburg.

### Comparison between countries

**Figure 1:**
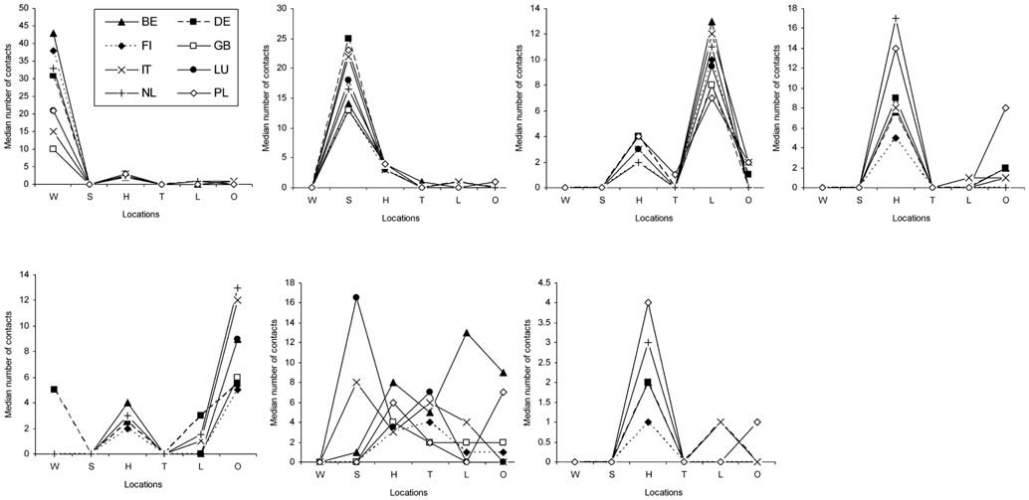
Distribution of contacts across locations for each contact profile by country (W = work, S = school, H = home, T = transport, L = leisure, O = other place).

The distribution of respondents across the seven contact profiles was roughly similar for all countries (Table 3). The *low contacts profile* indicating people with a low overall number of contacts was found most often in all of the countries (32–59%). Least stable was the *mixed profile* with contacts in different settings which was often found in Poland but was either absent or substantially smaller in other countries. Conversely, the profile indicating mainly contacts in not specified places (*other place profile*) was absent in Poland and infrequent in Germany. Additionally, there was substantial variability in the *big home* profile with a high number of contacts at home: it was found rarely in Germany, Netherlands and Poland. Less variation was found in the occurrence of the *professional profile*, *school profile* and *leisure profile*. The *school profile* was least frequent in Belgium and after checking the data it was explained by school holidays captured partly in the study period (Figure 1).

**Table 3:** Distribution of contact profiles across countries (%)*.

Profile	BE	DE	FI	GB	IT	LU	NL	PL
	N = 750	N = 1341	N = 1006	N = 1012	N = 849	N = 1051	N = 269	N = 1012
Professional	9.7	16.4	11.3	12.8	14.0	10.1	13.4	10.7
School	7.5	13.7	9.9	12.7	14.7	13.4	11.9	10.0
Leisure	7.2	5.7	13.3	8.0	13.5	12.0	11.9	6.8
Big home	8.9	4.5	15.8	10.6	14.0	7.2	2.6	4.0
Other place	8.4	.3	12.4	9.1	8.1	10.8	5.2	0
Mixed	.1	0	3.1	6.0	3.9	1.9	0	12.9
Low contacts	58.1	59.4	34.1	40.7	31.7	44.5	55.0	55.6

* based on most frequent classification in 100 runs with different ordering of cases

**Figure 2:**
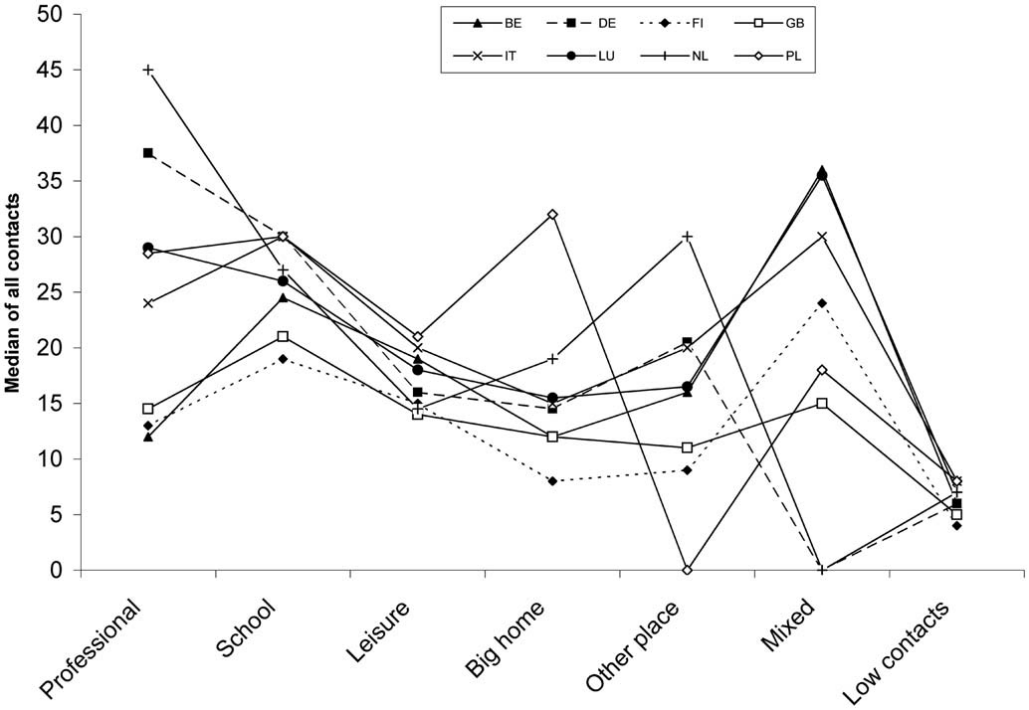
Median numbers of contacts for each cluster by country.

With regard to the number of contacts associated with each profile across countries there were strong similarities for the profiles: *school profile*, *leisure profile*, and *low contacts profile*. There was clearly more heterogeneity for *professional profile* and *mixed profile* (Figure 2). While the *other place profile* was absent in Poland, *big home profile* had substantially higher number of contacts, maybe indicating that contacts located in *other place* in other countries occur at home in Poland.

### Contact profiles and their characteristics

A contact profile describes how a respondent distributed his/her contacts across different settings on a particular day. Note that this might depend on the day of the week, so one person might have different contact profiles on different days of the week. The population is clearly grouped into clusters of people most of which have one main location for making contacts. This main location then dominates the contact behaviour (in terms of numbers of contacts) of that group. This is strongest for respondents with a *professional profile* and a *school profile*.

**Figure 3:**
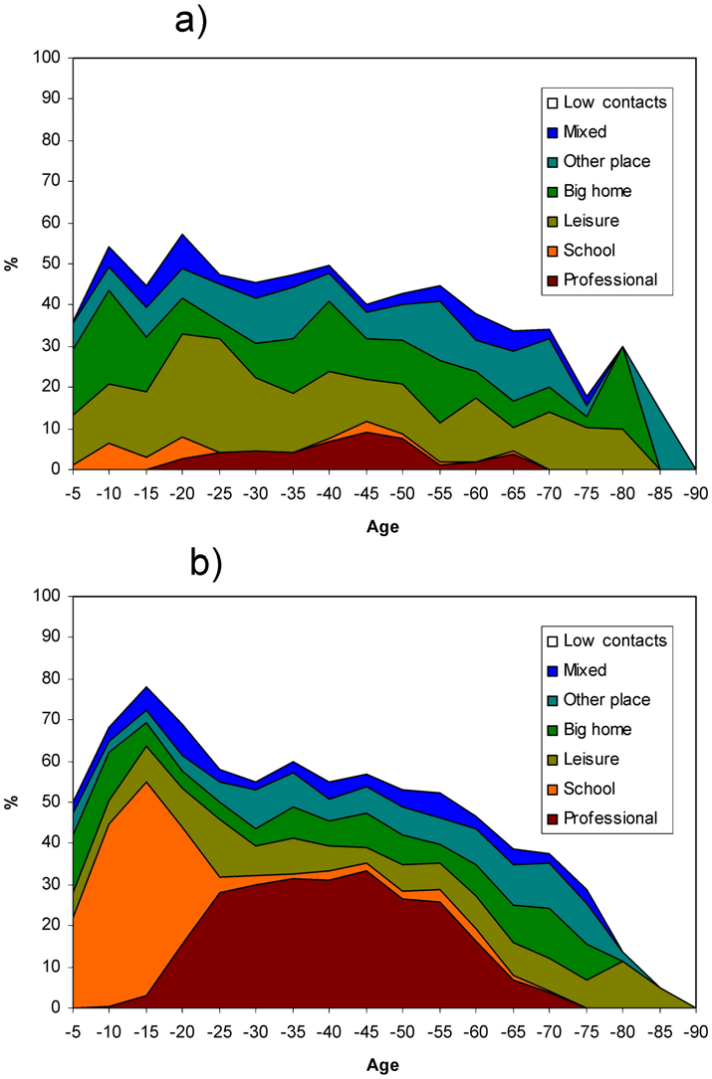
Distribution of contact profiles by age and weekdays (%). a) weekend. b) weekday.

As could be expected the *professional profile* was only found for respondents aged >15 yrs and was much more frequent during working days (Figure 3). The *school profile* was found mainly for respondents below age 20 and again mainly on working days. In contrast, the *leisure profile* was found across all age groups and more frequently during weekends. The *low contacts profile* was slightly more frequent on weekends and on both ends of the age spectrum.

**Figure 4:**
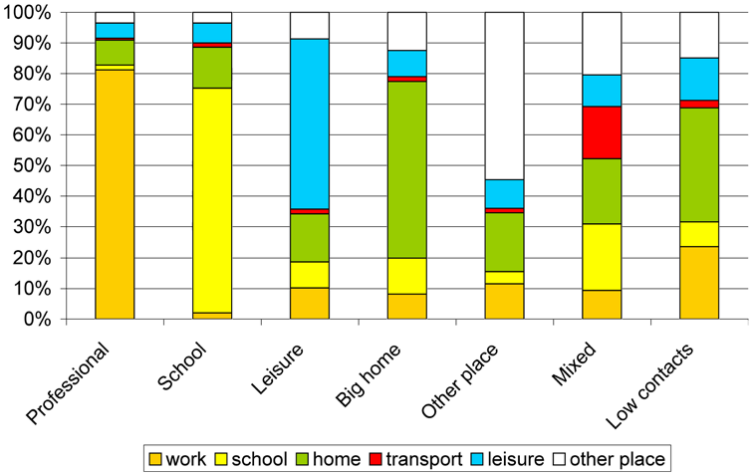
Distribution of contacts in different locations across the contact profiles.

Besides the different overall numbers of contacts in each profile, the fractions of contacts in different settings were different (Figure 4 presents data merged across all countries). While in each profile one setting dominates in terms of numbers of contacts occurring there, in all profiles the contacts at home play a significant role (Figure 4).

## Discussion

We analysed the way individual respondents in large population based surveys of conversational contacts distribute these contacts across different locations. We identified 7 different contact profiles on the basis of a cluster analysis of different individual contact distributions. In the evaluation of individual contact distributions in a study of 8 countries, we identified seven These distinct contact profiles were based on locations where the contacts take place: respondents having (1) mixed profile: contacts predominantly at school, during transportation and leisure time, (2) contacts during leisure time, (3) contacts mainly in the household (large family), (4) contacts at work, (5) contacts solely at school, (6) contacts in other places and finally (7) respondents having a low number of contacts in any setting. These profiles displayed a similar distribution in various European countries. There was a distinct age-dependence in the distribution of the population across contact profiles. In contrast to the earlier analysis in [4] where the distribution of all contacts across locations was analysed, our analysis provides insight into how individuals distribute their contacts across locations. In this way, our analysis provides new insight into the heterogeneity of contact patterns in populations and on the possible role of different contact locations for the overall connectedness of the population.

The analysis reveals patterns of behaviour that relate to different life styles and age groups, such as life styles dominated by work or school or life styles characterized by mainly household contacts. In all profiles except the “mixed” profile, one location where contacts take place dominates, but all profiles contain a certain fraction of household contacts. A large proportion of respondents belong to the cluster with low numbers of contacts in every location, but this might also include respondents who underreported their contacts. The distribution of respondents across profiles was remarkably similar for the eight countries included in the analysis.

The final number of the seven profiles might not be decisive, and in analyses conducted for specific purposes one might decide to use either higher or lower number of profiles. When a higher number of profiles is prescribed, some of the profiles described here will be split into new subunits: for example within the school profile we might have two groups, one with lower and one with higher average number of contacts. On the contrary, if the prescribed number of profiles is reduced some of the profiles will be merged, for example the mixed, leisure time and other place might be joined. Our choice of seven profiles is useful, because of its clear interpretation and the similar distribution across the analysed countries.

Limitations of the data collection and sampling are discussed in [4], we only summarize some important points here. Although the surveys in all countries attempted representative sampling, determinants as level of education, working hours and socio-economic class, that are known to affect participation in surveys, might also have an impact on the representativity of our findings. Since recruitment in most of the participating countries was based on household as a unit the results are not representative on the level of each country population. The methods of recruitment also slightly differed between the participating countries, which might have affected the distribution of household sizes. A major difficulty with the data set lies in the difference of data collection with respect to contacts at work. In some countries contacts at work and in Germany also contacts at school were recorded only as aggregate numbers if they exceeded a certain threshold. This means that for those countries it was not known whether respondents who filled in the questionnaire on a weekend day also had their reported work contacts on that weekend. In our analysis we attributed these contacts to working days. In Germany, where school contacts were recorded by asking for the size of the school class only, no information was available about contacts with individual class mates. This led to higher numbers of school contacts on average than recorded in other countries, where respondents only reported actual conversational contacts that had taken place with classmates. We did not have information about the category of contacts that were made at “other places”. Respondents might have differed in the way they attributed contacts to different categories, e.g. in what they considered leisure or other place.

The cluster analysis method we used is in some respects not optimally suited for the type of data that was analysed. Distributions of numbers of contacts are discrete distributions and usually display a high degree of overdispersion with some persons having large numbers and many persons having low numbers of contacts. These distributions are best described by negative binomial distributions (possibly shifted to the right) [3]–[5]. The distributions analysed here consisted of vectors of variables, of which some entries had a large mass at zero and others had a large mass away from zero. Therefore, the clustering algorithm subdivided the data set into clusters that are mainly characterized by subdivisions along the main coordinate axes. Possibly, more information could be extracted from the data by designing clustering algorithms that better take the specific structure of the data into account.

The results of our analysis provide guidance on the further development of mathematical models for the spread of airborne infectious diseases. While in [4] the emphasis was on mixing patterns amongst different age groups, here we demonstrate the importance of location as an environment for social contacts. Persons, who have contacts mainly at work have their contacts with other working people, persons who have contacts mainly at home meet a different strata of the population. Furthermore, there are “bridge locations” where people from different clusters meet and contact each other. Some modelling approaches have incorporated population structure that can account for contacts in different geographical locations [17] or in different social environments, e.g. [7], [18], [19], but not much empirical data has been available for parameterizing these models. Our results indicate that a stratification of the population into a limited number of subgroups with different contact profiles might be a parsimonious description of contact behaviour with respect to social environments.

Our results also have implications for designing control strategies for infections spread by close contact, e.g. respiratory infections and childhood infections. The shapes and distributions of various contact profiles show which part of a population will be affected by control measures intended to increase social distances. What is more, it also shows the connectivity between different subpopulations in the form of bridges between them, because persons with a given contact profile distribute their contacts in a specific way across locations. People with all contact profiles meet within their homes, which in that way not only provides a location for contacts with close proximity and long duration, but also serves as nodes where people with different contact profiles interact. It is not obvious what the impact of this bridge function is for transmission dynamics. Up to now, households have mainly been investigated as locations for within household transmission [20], but not in their function as bridges between population subgroups.

In conclusion, we have shown that although mixing by age remains the most important determinant of contact patterns, other structures emerge in the analysis of contact data that subdivide the population into groups with different patterns of behaviour. These patterns are linked to the major focus of activity in peoples' lives, but also demonstrate how different activities link different population groups via social contacts. Mathematical modelling studies can be designed that investigate the impact of these contact profiles and the way they link age groups and contact locations on the spread and control of infectious diseases.
